# Genomic Encyclopedia of Bacteria and Archaea: Sequencing a Myriad of Type Strains

**DOI:** 10.1371/journal.pbio.1001920

**Published:** 2014-08-05

**Authors:** Nikos C. Kyrpides, Philip Hugenholtz, Jonathan A. Eisen, Tanja Woyke, Markus Göker, Charles T. Parker, Rudolf Amann, Brian J. Beck, Patrick S. G. Chain, Jongsik Chun, Rita R. Colwell, Antoine Danchin, Peter Dawyndt, Tom Dedeurwaerdere, Edward F. DeLong, John C. Detter, Paul De Vos, Timothy J. Donohue, Xiu-Zhu Dong, Dusko S. Ehrlich, Claire Fraser, Richard Gibbs, Jack Gilbert, Paul Gilna, Frank Oliver Glöckner, Janet K. Jansson, Jay D. Keasling, Rob Knight, David Labeda, Alla Lapidus, Jung-Sook Lee, Wen-Jun Li, Juncai MA, Victor Markowitz, Edward R. B. Moore, Mark Morrison, Folker Meyer, Karen E. Nelson, Moriya Ohkuma, Christos A. Ouzounis, Norman Pace, Julian Parkhill, Nan Qin, Ramon Rossello-Mora, Johannes Sikorski, David Smith, Mitch Sogin, Rick Stevens, Uli Stingl, Ken-ichiro Suzuki, Dorothea Taylor, Jim M. Tiedje, Brian Tindall, Michael Wagner, George Weinstock, Jean Weissenbach, Owen White, Jun Wang, Lixin Zhang, Yu-Guang Zhou, Dawn Field, William B. Whitman, George M. Garrity, Hans-Peter Klenk

**Affiliations:** 1 DOE-Joint Genome Institute, Walnut Creek, California, United States of America; 2 Department of Biological Sciences, Faculty of Science, King Abdulaziz University, Jeddah, Saudi Arabia; 3 Australian Centre for Ecogenomics Research, School of Chemistry and Molecular Biosciences, The University of Queensland, Brisbane, Australia; 4 University of California, Davis, Davis, California, United States of America; 5 DSMZ - German Collection of Microorganisms and Cell Cultures GmbH, Braunschweig, Germany; 6 NamesforLife, LLC, East Lansing, Michigan, United States of America; 7 Max Planck Institute for Marine Microbiology, Bremen, Germany; 8 American Type Culture Collection (ATCC), Manassas, Virginia, United States of America; 9 Los Alamos National Laboratory, Bioscience Division, Los Alamos, New Mexico, United States of America; 10 School of Biological Sciences and Chunlab Inc., Seoul National University, Seoul, Korea; 11 University of Maryland, College Park, College Park, Maryland, United States of America; 12 Johns Hopkins Bloomberg School of Public Health, Johns Hopkins University, Baltimore, Maryland, United States of America; 13 AMAbiotics SAS, Genopole, Evry, France; 14 Ghent University, Department of Applied Mathematics and Computer Science, Ghent, Belgium; 15 Centre for Philosophy of Law, Université catholique de Louvain, Louvain-la-Neuve, Belgium; 16 Department of Civil and Environmental Engineering and Department of Biological Engineering, Massachusetts Institute of Technology, Cambridge, Massachusetts, United States of America; 17 Ghent University, BCCM/LMG Bacteria collection, Laboratory of Microbiology, Ghent, Belgium; 18 University of Wisconsin-Madison, Great Lakes Bioenergy Research Center, Madison, Wisconsin, United States of America; 19 Bioresource Center (BRC) of Institute of Microbiology, Chinese Academy of Sciences, P. R. China; 20 Institut National de la Recherche Agronomique, Jouy en Josas, France; 21 Institute for Genome Sciences, University of Maryland School of Medicine, Baltimore, Maryland, United States of America; 22 Human Genome Sequencing Center, Baylor College of Medicine, Houston, Texas, United States of America; 23 Institute for Genomics and Systems Biology, Argonne National Laboratory, Argonne, Illinois, United States of America; 24 BioEnergy Science Center (BESC), Oak Ridge National Laboratory, Knoxville, Tennessee, United States of America; 25 Jacobs University Bremen gGmbH, Bremen, Germany; 26 Lawrence Berkeley National Laboratory, Berkeley, California, United States of America; 27 Joint BioEnergy Institute (JBEI), Berkeley, California, United States of America; 28 Howard Hughes Medical Institute and Department of Chemistry and Biochemistry, University of Colorado, Boulder, Colorado, United States of America; 29 ARS, USDA, National Center for Agricultural Utilization Research, Peoria, Illinois, United States of America; 30 Theodosius Dobzhansky Center for Genome Bioinformatics, St. Petersburg State University, St. Petersburg, Russia; 31 Algorithmic Biology Lab, St. Petersburg Academic University, St. Petersburg, Russia; 32 Korean Collection for Type Cultures (KCTC), Korea Research Institute of Bioscience and Biotechnology (KRIBB), 111 Gwahangno, Yuseong-gu, Daejeon, Korea; 33 The Key Laboratory for Microbial Resources of the Ministry of Education, Kunming, People's Republic of China; 34 China General Microbiological Culture Collection Center (CGMCC), Institute of Microbiology, Chinese Academy of Sciences, Beijing, P. R. China; 35 CCUG - Culture Collection University of Gothenburg, Sahlgrenska Academy of the University of Gothenburg, Gothenburg, Sweden; 36 Diamantina Institute, The University of Queensland, St Lucia, Queensland, Australia; 37 Mathematics and Computer Science Division, Argonne National Laboratory, Argonne, Illinois, United States of America; 38 The J. Craig Venter Institute, Rockville, Maryland, United States of America; 39 Riken Bioresource Center, Japan Collection of Microorganisms, Hirosawa, Wako, Saitama, Japan; 40 Chemical Process & Energy Resources Institute, Centre for Research & Technology, Thessalonica, Greece; 41 Donnelly Centre for Cellular & Biomolecular Research, University of Toronto, Toronto, Ontario, Canada; 42 Department of Molecular, Cellular and Developmental Biology, University of Colorado, Boulder, Colorado, United States of America; 43 The Pathogen Genomics, The Wellcome Trust Sanger Institute, Hinxton, Cambridge, United Kingdom; 44 State Key Laboratory for Diagnosis and Treatment of Infectious Disease, The First Affiliated Hospital, College of Medicine, Zhejiang University, Hangzhou, China; 45 Institut Mediterrani d'Estudis Avançats (IMEDEA, CSIC-UIB), Esporles, Illes Balears, Spain; 46 CABI, Bakeham Lane, Egham, Surrey, United Kingdom; 47 Josephine Bay Paul Center for Comparative Evolution and Molecular Biology, MBL, Woods Hole, Massachusetts, United States of America; 48 Red Sea Research Center, King Abdullah University of Science and Technology (KAUST), Thuwal, Kingdom of Saudi Arabia; 49 NITE Biological Resource Center (NBRC), Kisarazu-shi, Chiba, Japan; 50 Department of Microbiology and Molecular Genetics, Michigan State University, East Lansing, Michigan, United States of America; 51 Department of Microbial Ecology, University of Vienna, Vienna, Austria; 52 The Jackson Laboratory for Genomic Medicine, Farmington, Connecticut; 53 Commissariat à l'Energie Atomique (CEA), Genoscope, Evry, France; 54 Department of Biology, University of Copenhagen, Copenhagen, Denmark; 55 Chinese Academy of Sciences Key Laboratory of Pathogenic Microbiology and Immunology, Institute of Microbiology, Chinese Academy of Sciences, Beijing, P. R. China; 56 U.K. Natural Environment Research Council (NERC), Environmental Bioinformatics Centre, Oxford, United Kingdom; 57 Department of Microbiology, University of Georgia, Athens, Georgia, United States of America

## Abstract

Microbes hold the key to life. They hold the secrets to our past (as the descendants of the earliest forms of life) and the prospects for our future (as we mine their genes for solutions to some of the planet's most pressing problems, from global warming to antibiotic resistance). However, the piecemeal approach that has defined efforts to study microbial genetic diversity for over 20 years and in over 30,000 genome projects risks squandering that promise. These efforts have covered less than 20% of the diversity of the cultured archaeal and bacterial species, which represent just 15% of the overall known prokaryotic diversity. Here we call for the funding of a systematic effort to produce a comprehensive genomic catalog of all cultured Bacteria and Archaea by sequencing, where available, the type strain of each species with a validly published name (currently∼11,000). This effort will provide an unprecedented level of coverage of our planet's genetic diversity, allow for the large-scale discovery of novel genes and functions, and lead to an improved understanding of microbial evolution and function in the environment.

## Abstract 2

This manuscript calls for an international effort to generate a comprehensive catalog from genome sequences of all the archaeal and bacterial type strains.

## Charting a New Path for Microbial Research

Earth is a microbial planet. Through their vast command of metabolic and catabolic processes, microorganisms control and sustain all life on Earth. They have no equal in their ability to survive in hostile environments or adapt to changing environmental conditions. By most any measure, microbes dominate the planet. Without them, life as we know it would cease to exist. They are our past—holding the secrets to the origins of life—and our future—sustaining life by maintaining essentially all of the biogeochemical cycles.

Yet we know surprisingly little about microbes. Today, we have the tools to make major advances in our understanding of how life evolves and functions in diverse habitats by determining the genome sequence of representatives of every known life form. Toward this goal, researchers are systematically targeting plant and animal species to fill in evolutionary gaps in the branches of the Tree of Life (ToL) (http://tolweb.org/tree/). However, these larger life forms constitute only a small portion of the tree and, being a relatively recent evolutionary innovation, represent only the last 550 million years of the more than 3,500,000,000 years of biological evolution on Earth. The great majority of the branches in the ToL are microbial, comprising the Bacteria, Archaea, protists, fungi, and viruses [1]–[5]. Even with 150 years of microbiological research completed, in which many of the major innovations have taken place over the past six decades, most of the microbial world—and therefore of biology as a whole—remains unexplored [6]–[10].

The first 15 years of microbial genome sequencing (1995–2009) yielded more than 1,000 complete genome sequences and another 1,000 draft genomes of Bacteria and Archaea [11]–[13]. Most of these projects were initiated based on potential practical applications for the selected organism, often in the fields of medicine (e.g., pathogens, drug targets, and probiotics) or biotechnology (e.g., biopharmaceuticals, bioenergy, agriculture, environmental remediation, and industrial production of microbial products). While this application-driven science provided a significant gain in information for those purposes, it ignored most of the microbial diversity on the planet [1],[7],[9]–[10]. It is time to move beyond this approach to launch a systematic genomic exploration of all validly named microbial species, starting (for pragmatic reasons based on genome size) with Bacteria and Archaea. The goal of this ambitious but, given the currently available technologies, assuredly tractable initiative is to sequence the genome of at least one representative of every bacterial and archaeal species whose name has been validly published in accordance with the International Code of Nomenclature of Bacteria (Bacteriological Code) [14]–[19].

Each of these approximately 11,000 bacterial and archaeal species has a designated type strain, a living culture that serves as a fixed reference point for the assignment of bacterial and archaeal names, thus often also denoted as a reference strain (see Box 1). A type strain is not the archetypal representative of a species, a common misperception. Thus, type strains play a crucial role in defining the phylogenomic and taxonomic space of Bacteria and Archaea, facilitating efforts to assign evolutionary relationships and identify new species. By definition, type strains are descendants of the original isolates used in species and subspecies descriptions, as defined by the Bacteriological Code [14], that exhibit all of the relevant phenotypic and genotypic properties cited in the original published taxonomic circumscriptions. They are made available in pure culture (except in cases such as symbionts) for subsequent taxonomic revision in accordance with the rules defined by the Bacteriological Code [14],[17]–[19]. The type strains represent the only area of the microbiological sciences in which the deposit and availability of biological material is mandatory, allowing the verification of past work and potentially extending to further aspects as time and technology become available by using the same biological material.

**Box 1. The Value of Type and Reference Strains**

Genomic information from a limited sampling of type strains can refine our understanding of the breadth and depth of the phylogenetic space known from previously published taxonomic studies. The synergy between classification and genomics [31] could catalyze an enhanced view and understanding of those microorganisms, as outlined in a recent American Academy of Microbiology (AAM) report [32]. Similarly, the GEBA project will aim to fully cover a defined portion of the extant diversity by targeting the approximately 11,000 type strains that represent the complete current list of Bacteria and Archaea with validly published names. Given that the richest metadata is associated with the type strains, a focused, in-depth survey such as this will offer significant benefits by providing genomic data to complement the wealth of information already acquired for these organisms. The metadata, such as the physiology of the organism, will reciprocate by validating the genome-based metabolic reconstructions.

To keep track of the constantly increasing number of type strains for Bacteria and Archaea and the list of those that have a genome sequencing project, the Microbial Earth Project (MEP) was recently launched. MEP (http://www.microbial-earth.org/) is a public resource providing frequently updated information on the status of sequencing coverage of the type strains. The resource, maintained at the DOE Joint Genome Institute, provides data based on the type-strain information available from N4L (http://namesforlife.com/) and genome projects available from GenomesonLine Database (http://www.genomesonline.org/) [13]. MEP displays the list of type strains with and without genome sequencing projects as a list or as an interactive map (Figure 2).

**Figure 1:**
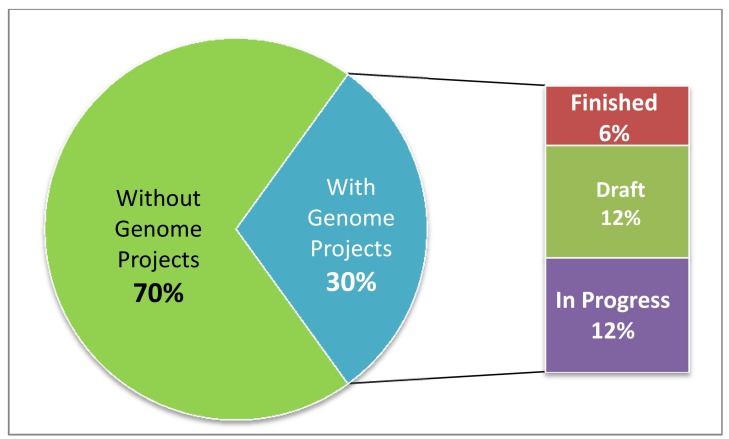
Genome project coverage of bacterial and archaeal type strains. From a total of approximately 11,000 bacterial and archaeal type strains, 3,285 (30%) have a publicly known genome project.

Of the approximately 25,000 documented bacterial and archaeal genome projects [13], 3,538 target 3,285 nonredundant type strains out of the currently estimated 11,000 (30%) (Figure 1; Table 1). If we continued this largely application-driven mode of selecting sequencing targets, another 83,000 genome projects would be required in order to cover the type strains for the 11,000 species that represent the part of the cultivated diversity of Bacteria and Archaea with validly published names. Despite the comparatively low funding support for taxonomic work, about 650 new species names are validly published per year (according to the rules defined by the Bacteriological Code), pointing to an ever-increasing gap.

**Figure 2:**
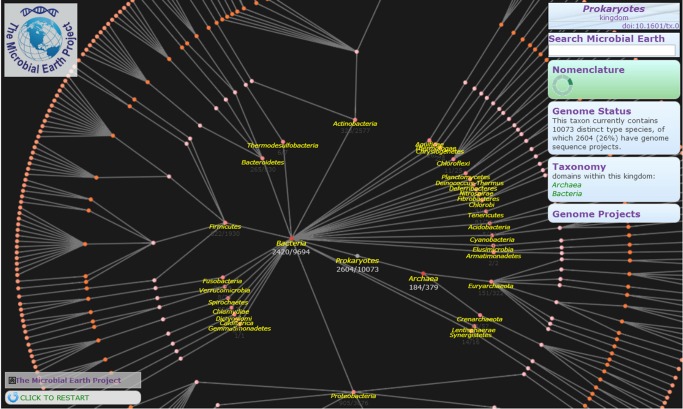
Interactive map based on the NamesforLife (N4L) taxonomic information of the type strains. Each leaf represents a type strain. Colors denote strains with or without genome projects. Lighter colored nodes denote higher taxonomic ranks. Branch lengths are not meaningful.

**Table 1:** Numbers of Archaea and Bacteria.

number of nonredundant 16S rRNA genes from Bacteria and Archaea	479,726^1^
number of cultured Bacteria and Archaea	Unknown
number of cultured Bacteria and Archaea available in culture collections	106,372^2^
number of cultured Bacteria and Archaea in culture collections that are type strains	∼11,000^3^^,^ 4
number of cultured Bacteria and Archaea in culture collections that are type strains and have a genome sequencing project	3,285^5^
number of cultured Bacteria and Archaea in culture collections that are type strains and have a genome sequencing project at finished or draft stage	1,964^5^
number of Bacteria and Archaea strains with genome projects	24,559

1 http://www.arb-silva.de;

2 http://wdcm.org;

3 http://services.namesforlife.com/home;

4 http://www.bacterio.cict.fr;

5 http://genomesonline.org/.

Therefore, the first phase of the proposed effort should systematically target the 7,830 type strains not previously addressed for high-quality draft genome sequencing [20]. Finishing a high-quality draft sequence should be targeted for at least one representative of each genus, with the type strain of the type species having priority [21]. Simultaneously, type strains of all new species and subspecies whose names are validly published should be sequenced at the time they are deposited into culture collections. As ongoing technological advances continue to reduce sequencing costs, sequencing and publication of the genome, which is already far simpler than phenotypic characterization, will become a routine part of the strain deposition process.

## Closing the Phylogenetic Gap

Previously, microbial genome sequencing projects were initiated primarily by individual researchers who targeted one or a few microorganisms of interest. With the advent of new high-throughput sequencing technologies, we are witnessing a shift from “one principal investigator (PI), one genome” projects to large-scale sequencing initiatives that engage a wider research community. Cataloging Earth's microbial genetic diversity cannot realistically be achieved by a single sequencing center, a single culture collection, a single funding agency, or even a single country. International cooperation—to share both the work and its funding—will be essential. The study and understanding of microbial life—and for that matter, all life—cannot be separated or divided by man-made silos based on application or economic relevance. Indeed, we have reached the point at which scientific progress can be hindered and limited by the insulation of individual funding agencies.

While prospects for developing a groundbreaking interagency funding mechanism remain on the horizon, efforts to forge multinational collaborations are underway. A consensus agreement has already been achieved among some of the major sequencing facilities and culture collections in the United States, Europe, and Asia that will lead the DNA isolation and sequencing efforts.

The time is ripe for a cooperative venture of this scale. High-profile examples of such successfully coordinated efforts include the pilot project of the *Genomic Encyclopedia of Bacteria and Archaea* (GEBA) (http://www.jgi.doe.gov/programs/GEBA/) and the Human Microbiome Project (HMP) (http://www.hmpdacc.org/).

The US Department of Energy (DOE)-funded pilot GEBA project is the first large-scale effort applying phylogenetically balanced sampling of the bacterial and archaeal branches of the ToL. Its goal, the sequencing of 250 microbial genomes selected based on their phylogenetic novelty, required a coordinated pipeline for microbial cultivation and DNA extraction, sequencing, annotation, and comparative analysis. The publication of the first 56 draft genomes from this project [22] confirmed that vast uncharted genetic novelty does in fact exist in nature. Gaining a deeper understanding of that genetic novelty demands the systematic genomic characterization of ultimately all bacterial and archaeal species across the ToL. Toward that end, the CyanoGEBA project took a phylum-level approach to sequence 54 phylogenetically and phenotypically diverse strains of cyanobacteria [23]. More recently, the aptly named GEBA-Microbial Dark Matter (GEBA-MDM) (http://genome.jgi.doe.gov/MDM/MDM.home.html) explored the diversity of the vast universe of uncultured microbes by using high-throughput single-cell sequencing to generate a reference dataset of 201 single-cell genomes from candidate phyla [24]. At the same time, these initiatives have also stimulated the quest for novel organisms in these previously uncultivated groups, further increasing the number of strains available for study.

The National Institutes of Health (NIH)-funded HMP project broke new ground in microbial genomics by virtue of the unprecedented volume of sequence data generated by sequencing approximately 1,000 microbial genomes [25]. Of even greater consequence is the distribution of the work across several large-scale sequencing facilities (i.e., the J. Craig Venter Institute, Washington University, Baylor College of Medicine, and the Broad Institute). By organizing the project in this manner—a style reminiscent of the human genome effort—the NIH created a timely opportunity for collaboration among some of the world's leading sequencing and analysis centers, thus in effect mandating the standardization of their sequencing, finishing, and analysis pipelines. Furthermore, an International Human Microbiome Consortium (IHMC) (http://www.human-microbiome.org/) was formed to coordinate the activities and policies of the individual international groups and to facilitate the work under a common set of principles and policies.

We are also seeing individual sequencing centers scale up their throughput capacity dramatically. For example, the Beijing Genomics Institute (BGI) announced a project, in conjunction with several other institutions, to draft sequence the genomes of 10,000 Chinese microbial isolates in 3 years. More recently, the Sanger Institute has announced plans to sequence 3,000 type strains from the United Kingdom (UK)'s National Collection of Type Cultures (NCTC) and make them available as a community resource. Overall, these large-scale initiatives confirm that our proposed project is well within the current international sequencing capacity. Indeed, even if one forecasts a conservative linear increase in the number of genome projects, one would expect to see at least 20,000 strains sequenced in the next 2–3 years [20]. The real challenge now is to create a global collaboration that can productively channel this capacity by guiding the selection of genome projects, eliminating redundancies, and establishing international standards [26].

## Standards for Success

As the HMP project has already shown, a widely distributed international project can only succeed if uniform standards are developed and agreed upon at the beginning and if all participants then adhere to them throughout the project (see Box 2). To this end, we propose that such an effort will be conducted in close collaboration with the Genomics Standards Consortium (GSC) [26], which has been spearheading the international effort to define standards for sequencing and analysis [20],[26]–[29]. At the same time, the involvement of culture collections that have helped to shape recent Organization for Economic Cooperation and Development (OECD) Biological Resource Centre (BRC)-oriented documents and the taxonomic infrastructure surrounding the International Committee on Systematics of Prokaryotes (ICSP) and the Bacteriological Code will ensure that established standards are also integrated to create a comprehensive and authoritative output.

**Box 2. Global Data Standards**

Accurate estimates of diversity will require not only standards for data but also standard operating procedures for all phases of data generation and collection [33],[34]. Indeed, sequencing all archaeal and bacterial type strains as a unified international effort will provide an ideal opportunity to implement international standards in sequencing, assembly, finishing, annotation, and metadata collection, as well as achieve consistent annotation of the environmental sources of these type strains using a standard such as minimum information about any (X) sequence (MixS) [27],[29]. Methods need to be rigorously challenged and validated to ensure that the results generated are accurate and likely reproducible, without having to reproduce each point. With only a few exceptions [27],[29], such standards do not yet exist, but they are in development under the auspices of the Genomics Standards Consortium (e.g., the M5 initiative) (http://gensc.org/gc_wiki/index.php/M5) [35]. Without the vehicle of a grand-challenge project such as this one, adoption of international standards will be much less likely.

Within the culture collection community, significant progress has been made in the creation of working documents produced as part of OECD-based initiatives [32],[36]. Most of these reflect established working practices in the more prominent collections and will serve as the basis for the long-term availability of the strains that will constitute the core of this project.

Technological developments within taxonomy have also ensured that an ever-increasing spectrum of parameters is taken into consideration, providing a complementary source of information on the expressed properties of the organisms concerned [16]. These serve as international standards in the way organisms are characterized at this level. The requirement that type strains be deposited in two collections in two different countries also ensures long-term availability of this biological reference material, as well as introducing a verification step during the process of accession. The synergy of these three elements will provide an unprecedented set of standards that will serve to significantly improve the quality of the data obtained.

Such transformation of the existing research infrastructure into a globally distributed and digitally integrated network for microbial research, including computational science and automated knowledge discovery, would require overcoming obsolete and science-hostile database protection laws as well as highly restrictive licensing practices of biological materials [37]. Therefore, all essential public knowledge assets and the results of the proposed effort would be linked into a global microbial research commons and thus available to the scientific community, without restrictions to the fullest extent possible. The proposed research commons would enable qualified participants to contractually override the legal obstacles and access a digitally integrated, ever-expanding pool of biological materials, sequence data, and associated literature [37],[38].

The implementation of accepted community standards for this international project will be accompanied by an international educational outreach program to provide training and support to undergraduates and postgraduates and to promote widespread implementation of these standards for sequencing and analysis.

Any project of this scale and breadth depends on harnessing existing knowledge and resources to succeed. By focusing on the type and other reference strains of Bacteria and Archaea, the GEBA project will build on the wealth of experimental knowledge and metadata already acquired for these organisms. A further advantage is that these strains are already available to the global research community and are stored in professional units that are dedicated to long-term storage and distribution. Adding the genomic component will increase the value of that knowledge and will, in turn, be enriched by it. While completion of the GEBA project will leave much of the extant microbial diversity unexplored, its systematic sequencing would provide a core of more than 11,000 bacterial and archaeal type strains (including the additional species expected to be described)—a solid foundation that can inform the ongoing inquiry into microbial diversity in its entirety. This framework of high-quality genomes from well-characterized type strains is especially important in light of recent advances in genome recovery via culture-independent approaches, namely single-cell and population genomics, which are rapidly adding genomic foliage to the tree of life (see Box 3) [30]. Without this framework, the exploration of our microbial planet is equivalent to navigation without a compass, map, or stars by which to fix one's position.

**Box 3. Creating a Comprehensive Microbial Genomic Framework**

Although cultured microorganisms are commonly said to represent only ∼1% of the Earth's Bacteria and Archaea based on the difference between plate counts and observed cells [39]–[40], phylogenetic coverage offers a more meaningful metric. Using Faith's phylogenetic diversity (PD), i.e., unique branch length in small-subunit (SSU) rRNA trees as the metric [41], species with validly published names are estimated to account for 15.3% of the total bacterial and archaeal diversity known from SSU rRNA sequences obtained from Sanger sequencing—not an insignificant fraction. Currently recognized genome projects have mapped ∼2.8% of that known microbial diversity [13]. Sequencing all of the remaining type strains will increase the phylogenetic coverage encompassed and will then approach 15% of the known bacterial and archaeal diversity, thus expanding the framework on which rests the study of microbiology as a whole.

This expanded collection of sequenced genomes will be of great value as a reference library for the interpretation of metagenomic data [42] obtained from diverse microbial communities and from grand-scale surveys such as the European MetaHIT [43], the international Terragenome projects (http://www.terragenome.org/), and the Earth Microbiome Project [44], which is a natural extension of this effort. The prodigious quantity and fragmented nature of metagenomic sequences have prompted the development of new bioinformatics methods for their analysis. However, meaningful functional and taxonomic interpretation of metagenomic sequences requires a comprehensive library of reference genomes that encompass the extant evolutionary diversity so that the anonymous sequence fragments can be assigned a place within the ToL. Even the relatively modest increase in representation provided by the genomes sequenced so far by the GEBA, GEBA-MDM, and HMP projects has afforded more accurate classification of metagenomic data and thus more trustworthy interpretation of sequences from the human microbiome and other environments. By extrapolation, it is apparent that such an effort will significantly improve our ability to interpret metagenomic data.

The large-scale sequencing facilities that have spearheaded the genomics revolution in microbiology during the last decade, along with the biological research centers that capture and maintain Earth's cultured microbial diversity and the larger community of microbiologists, are now coming together to form an unparalleled and truly global initiative that promises to change the way we study microbial life. Only with such a massive undertaking can we hope to unlock the secrets underlying the evolutionary success of the smallest, most enduring organisms on Earth.
